# Transfusion Guidelines in Traumatic Brain Injury: A Systematic Review and Meta-Analysis of the Currently Available Evidence

**DOI:** 10.1089/neur.2022.0056

**Published:** 2022-12-22

**Authors:** Eric Y. Montgomery, Umaru Barrie, Yves J. Kenfack, Derrek Edukugho, James P. Caruso, Benjamin Rail, William H. Hicks, Emmanuella Oduguwa, Mark N. Pernik, Jonathan Tao, Paula Mofor, Emmanuel Adeyemo, Tarek Y. El Ahmadieh, Mazin Al Tamimi, Carlos A. Bagley, Nicole Bedros, Salah G. Aoun

**Affiliations:** ^1^ Department of Neurological Surgery, University of Texas Southwestern Medical Center, Dallas, Texas, USA.; ^2^ Department of Neurological Surgery, Boonshoft School of Medicine, Wright State University, Dayton, Ohio, USA.; ^3^ Department of Neurological Surgery, Memorial Sloan Kettering Cancer Center, New York, New York, USA.; ^4^ Department of Surgery, Baylor University Medical Center, Dallas, Texas, USA.

**Keywords:** anemia, hemoglobin, systematic review, TBI, transfusion, traumatic brain injury

## Abstract

Our study aims to provide a synthesis of the best available evidence on the hemoglobin (hgb) red blood cell (RBC) transfusion thresholds in adult traumatic brain injury (TBI) patients, as well as describing the risk factors and outcomes associated with RBC transfusion in this population. A systematic review and meta-analysis was conducted using PubMed, Google Scholar, and Web of Science electronic databases according to the Preferred Reporting Items for Systematic Reviews and Meta-Analyses (PRISMA) guidelines to assess articles discussing RBC transfusion thresholds and describe complications secondary to transfusion in adult TBI patients in the perioperative period. Fifteen articles met search criteria and were reviewed for analysis. Compared to non-transfused, TBI patients who received transfusion tended to be primarily male patients with worse Injury Severity Score (ISS) and Glasgow Coma Scale. Further, the meta-analysis corroborated that transfused TBI patients are older (*p* = 0.04), have worse ISS scores (*p* = 0.001), receive more units of RBCs (*p* = 0.02), and have both higher mortality (*p* < 0.001) and complication rates (*p* < 0.0001). There were no differences identified in rates of hypertension, diabetes mellitus, and Abbreviated Injury Scale scores. Additionally, whereas many studies support restrictive (hgb <7 g/dL) transfusion thresholds over liberal (hgb <10 g/dL), our meta-analysis revealed no significant difference in mortality between those thresholds (*p* = 0.79). Current Class B/C level III evidence predominantly recommends against a liberal transfusion threshold of 10 g/dL for TBI patients (Class B/C level III), but our meta-analysis found no difference in survival between groups. There is evidence suggesting that an intermediate threshold between 7 and 9 g/dL, reflecting the physiological oxygen needs of cerebral tissue, may be worth exploring.

## Introduction

With an estimated 69 million people worldwide and 2.8 million in the United States suffering from a traumatic brain injury (TBI) annually, TBI is a leading cause of disability and death.^1,2^ The pathophysiology of blunt TBI is characterized by a primary external blow to the brain causing visceral or ischemic injury, with a secondary cascade of inflammatory-related events that can further disrupt neuronal function.^3^ Anemia in the setting of TBI carries the risk of worsening cerebral ischemia, especially if associated with intracranial hemorrhage with increased intracranial pressure and diminished cerebral perfusion pressure. Red blood cell (RBC) transfusion is a common method of treating anemia, but transfusions can lead to other adverse events.^4,5^ Establishing a target transfusion threshold in TBI patients must balance the unique oxygen requirements of the brain and the potential complications of transfusion.

Some guidance exists from the TRICC (Transfusion Requirements in Critical Care) trial, which compared restrictive and liberal transfusion strategies in intensive care unit (ICU) patients.^6^ The study found no difference in 90-day mortality, with a hemoglobin transfusion threshold of 7 g/dL compared to a threshold of 10 g/dL. However, this trial was not limited to TBI patients. Two more recent randomized controlled trials (RCTs) have been performed, but have not provided definitive superiority of one strategy over the other.^7,8^ Optimized oxygen-carrying capacity is vital to prevent permanent neurological dysfunction after TBI, and the establishment of a TBI-specific transfusion threshold may help prevent the long-term consequences of persistent anemia. Therefore, the objective of this systematic review is to review current literature to better define the optimal transfusion threshold for TBI patients. We also evaluate the outcomes and complications associated with transfusions, along with the risk factors for requiring transfusion, in this population. This review aims to better equip neurosurgeons and critical care physicians with the most up-to-date evidence for the administration of RBC transfusions in the TBI population.

## Methods

### Search strategy

The search methodology was carried out in accordance with the Preferred Reporting Items for Systematic Reviews and Meta-Analyses (PRISMA) guidelines. Query of the medical literature using PubMed, Web of Science, and Google Scholar databases was conducted with keywords of “Transfusion” AND “Traumatic Brain Injury.” There was no time-frame limit to the search, and only studies pertaining to adults (≥18 years old) were included in the study. Exclusion criteria were as follows: pediatric patient population (<18 years old), penetrating TBI, blood-type analysis, studies that assessed blood-loss prevention strategies, technical studies, animal studies, patients with hematological malignancies, review articles, letters to the editor, meta-analyses, and non-English articles.

### Data extraction

Three authors (E.M., Y.K., and D.O.) independently performed the data extraction according to the aforementioned search strategy.^9^ Articles were initially screened by title and abstract. Full-text review was performed on relevant articles for extraction of pertinent data. The primary outcomes of the literature review were to 1) compare outcomes based on hemoglobin (hgb) threshold for RBC transfusion, 2) identify risk factors for receipt of RBC transfusion after TBI, and 3) evaluate differences in clinical outcomes and complications associated with receipt of transfusion. Data collected include study author, year of publication, study type, research aim, patient population, transfusion threshold recommendation, as well as characteristics at presentation such as age, sex, Glasgow Coma Scale (GCS), Injury Severity Score (ISS), and head Abbreviated Injury Scale (AIS). Follow-up outcomes of interest included mortality, length of stay (LOS), and Glasgow Outcome Scale (GOS; or Glasgow Outcome Scale-Extended [GOSE]). The GOS or GOSE is the gold-standard metric for outcomes in TBI patients, quantifying recovery by the degree of return to daily activities as well as residual disability.^10^ Three authors (S.G.A., J.P.C., and U.B.) provided guidance on any incongruencies between studies.

### Levels and classes of evidence

Levels and classes of evidence were determined based on the American College of Cardiology (ACC)/American Heart Association (AHA) evidence-based scoring system and are reported in Appendix A for each article.

### Meta-analysis

After identification of eligible studies, the following parameters were collected: study author, year of publication, overall study population size, number of patients who were not transfused, number of patients who were transfused, transfusion hgb threshold, age, GCS on presentation, ISS on presentation, mean number of packed RBC (pRBC) transfusions, complication rate, and mortality rate. When comparing patients who did and did not receive transfusions, the primary outcome measures were overall complications and mortality. When comparing patients who were transfused at liberal or restrictive thresholds, the primary outcome measures were number of pRBC transfusions and mortality.

Categorical variables were summarized as standardized mean differences (SMDs), and continuous variables were summarized as risk ratios, odds ratios, or log risk ratios, in accordance with effect measures reported in the included individual studies. When absent, confidence intervals and measures of standard error were derived using reported data according to standard methods outlined in the Cochrane Handbook. Study heterogeneity was calculated using Cochrane's *Q* and Higgins' *I*^2^ statistics, with *Q* statistic values within a 10% level of significance (*p* < 0.1) and *I*^2^ values >50% representing substantial heterogeneity. Given the variability in population characteristics and methodology between studies, we used an inverse variance random-effects model using the DerSimonian and Laird method for each comparison. Additionally, leave-one-out analysis was used to investigate each study's influence on pooled estimates and effect size for each comparison. Forest plots were created to represent effect sizes and pooled estimates. All statistical tests were two-sided, with *p* < 0.05 representing a statistically significant association.

## Results

### Electronic search yield

**FIG. 1.**
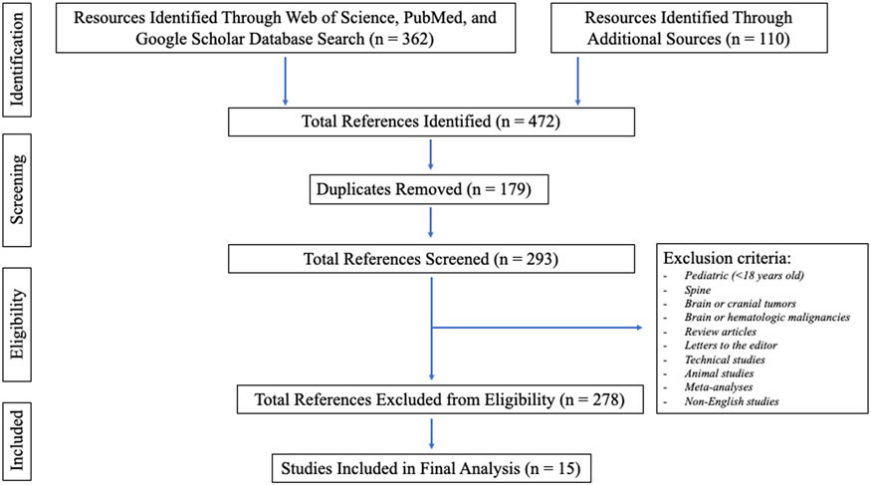
PRISMA flow diagram detailing the methodology of the systemic review. PRISMA, Preferred Reporting Items for Systematic Reviews and Meta-Analyses.

Our initial database search yielded 362 studies, with an additional 110 studies identified through additional sources (Fig. 1). One hundred seventy-nine duplicates were removed, and 278 studies did not meet inclusion criteria. A total of 15 articles were included in the final analysis. Appendix A presents a concise summary of those articles along with the corresponding ACC/AHA level and class of evidence. Six studies provided data on transfusion threshold recommendation (Table 1),^5,7,8,11–13^ seven on risk factors associated with receipt of transfusion (Table 2),^4,14–19^ and seven on transfusion-related complications (Table 3).^4,14–19^

**Table 1.** Studies Assessing the Association of Blood Transfusions Thresholds with Clinical Outcomes in TBI Patients

Study	Design	hgb threshold (g/dL)	Transfused patients:* N *(%)	Results							Transfusion threshold recommendations	AHA classification of evidence
				Total LOS (days): mean (SD or range)	ISS: mean (SD or range)	GCS: mean (SD or range)	AIS: mean (SD or range)	ARDS-free survival:* n *(%) or OR (95% CI)	Favorable GOS/GOSE:* n *(%) or OR (95% CI)	Death (*n*; %) or 28-day survival (OR +95% CI)		
Elterman et al., 2013^35^	Multi-center (USA, Canada)	<7	1158		Overall: 27.0 (15.6) Head: 3.5 (1.8)	5.2 (2.6)		OR: 0.98 (0.79–1.22)	0.88 (0.57–1.34)	OR: 0.95 (0.78–1.16)	Transfusion when initial hgb >10 is associated with decreased survival	Class B-R level III, risk
		7–10						OR: 1.00 (0.92–1.09)	0.92 (0.84–1.02)	OR: 0.99 (0.99–1.09)		
		>10						OR: 0.82 (0.74–0.92)	0.82 (0.71–0.94)	OR: 0.83 (0.74–0.93)		
Litofsky et al., 2016^36^	Single institution (USA)	<10	164		29.30	9.80	4.0		92 (56.0%)		Transfusion was associated with worse outcomes with an hgb threshold <9 or 10 g/dL and improved outcomes in patients with hgb <7 and 8 g/dL—consider the 8-g/dL threshold.	Class C-LD level III, risk
		<9	104		30.90	9.40	4.1		51 (49.0%)			
		<8	56		33.80	8.10	4.2		21 (38.2%)			
		<7	24		40.60	6.40	4.5		4 (17.4%)			
Ngwenya et al., 2018^37^	Single institution (USA)	<7	586	10.9 (13.6)	19.3 (11.6)	12 (3.8)				101 (10.3%)	Outcomes did not significantly differ according to transfusion threshold.	Class C-LD, level III, risk
		<10	979	13.9 (23.3)	18.1 (10.6)	12 (3.7)				49 (8.4%)		
Robertson et al., 2014^38^	Single institution (USA)	<7	99		29 (25–38)	GCS <8 57		83 (83.8%)	37 (42.5%)	14 (14.1%)	Outcomes did not significantly differ according to transfusion threshold.	Class B-R level III, risk
		<10	101		29 (25–35)	54		76 (75.2%)	31 (33.0%)	17 (16.8%)		
Gobatto et al., 2019^39^	Randomized controlled trial	<7	23	42 (23–76)	31 (9)	5 (3–7)	5 (3–7)		10 (43.5%)	7 (30.4%)	Survival and neurological outcomes were superior with a comparatively liberal transfusion threshold.	Class B-R level IIa, benefit
		<9	21	35 (21–63)	28 (9)	4 (3–7)	4 (3–7)		13 (61.9%)	2 (9.5%)		
McIntyre et al., 2006^40^	Subgroup analysis of randomized controlled trial	<7	29	27 (14–39)	29.8 (14.0)	7.3 (3.6)				5 (17%)	Mortality did not significantly differ according to transfusion threshold.	Class B-R level III, risk
		<10	38	20.5 (17–47)	31.3 (13.0)	7.5 (3.4)				5 (13%)		

TBI, traumatic brain injury; AIS, Abbreviated Injury Score; ARDS, acute respiratory distress syndrome; GCS, Glasgow Coma Score; GOS, Glasgow Outcome Score; GOSE, Glasgow Outcome Score-Extended; hgb, hemoglobin; ISS, Injury Severity Score; LOS, length of stay; SD, standard deviation; OR, odds ratio; CI, confidence interval; AHA, American Heart Association.

**Table 2.** Demographics, Comorbidities, and Intraoperative Characteristics of Transfused versus Non-Transfused TBI Patients in the Studies Included in the Meta-Analysis

Study	Transfusion status	Sample size (*n*)	Age (years): mean (SD) or median (IQR)	Female:* N *(%)	GCS:* N *(%) or mean (SD) or median (IQR)	AIS:* N *(%) or mean (SD) or median (IQR)	ISS: mean (SD) or median (IQR)
Boutin et al., 2017^41^	Non-transfused	5071	48.7	1271 (25.1%)	GCS <9 1283 (25.3%)	Head AIS >3 6682 (95.5%)	
	**Transfused**	**1991**		**623 (31.3%)**	**505 (25.4%)**	**1839 (92.4%)**	
Warner et al., 2010^42^	Non-transfused	63	40.9 (20.6)		9.7 (5.1)	Head 3.9 (0.8)	26.5 (9.1)
	**Transfused**	**76**	**39.8 (19.3)**		**7.1 (5.0)**	**4.0 (0.8)**	**29.8 (10.7)**
Boutin et al., 2018^43^	Non-transfused	149		21 (31.8%)	GCS <9 42 (28.2%)	Head AIS >3 142 (95.3%)	
	**Transfused**	**66**		**26 (17.4%)**	**18 (27.3%)**	**66 (100%)**	
Leal-Noval et al., 2016^44^	Non-transfused	145	40 (25–73)	17 (11.7%)	7 (6–10)	Head 4 (4–5)	20 (16–25)
	**Transfused**	**164**	**42 (30–60)**	**38 (23.2%)**	**9 (7–12)**	**5 (4–5)**	**29 (25–34)**
Al-Dorzi et al., 2015^45^	Non-transfused	80	28.6 (14.3)	5 (6.2%)	7.0 (3.5)	Total 24.3 (5.2)	19.9 (7.3)
	Transfused	**21**	**30.8 (14.8)**	**0 (0%)**	**6.0 (3.2)**	**23.3 (3.5)**	**22.0 (6.8)**
George et al., 2008^46^	Non-transfused	39	54.6 (23.9)	13 (33.3%)	5.5 (2.4)	Head 4.77 (0.54)	23.5 (4.5)
	**Transfused**	**43**	**52.6 (19.5)**	**14 (32.6%)**	**4.2 (1.7)**	**4.88 (0.5)**	**25.0 (3.3)**
Salim et al., 2008^47^	Anemic: non-transfused	126	36.8 (23.7)	43	10.5 (4.6)	Head AIS >3 56 (44%)	23.5 (4.5)
	**Anemic: transfused**	**401**	**42.8 (21.7)**	**111**	**9.1 (4.9)**	**208 (52%)**	**25.0 (3.3)**
	Not anemic: non-transfused	495	36.9 (19.2)	10	10 (4.5)	179 (36%)	23.5 (4.5)
	**Not anemic: transfused**	**128**	**45.1 (21.3)**	**43**	**8.1 (4.8)**	**73 (57%)**	**25.0 (3.3)**

TBI, traumatic brain injury; AIS, Abbreviated Injury Score; GCS, Glasgow Coma Score; ISS, Injury Severity Score; SD, standard deviation; IQR, interquartile range.

**Table 3.** Post-Operative Complications Associated with Transfused versus Non-Transfused TBI Patients in the Studies Included in the Meta-Analysis

Study	Transfusion status	Sample size (*n*)	Average total LOS (days): RR or mean (SD) or median (IQR)	Complications:* N *(%) or RR (95% CI)	Functional outcome:* N *(%) or mean (SD)	Death:* N *(%) or RR (95% CI)
Boutin et al., 2017^41^	Non-transfused	5071	(ref)	(ref)		(ref)
	**Transfused**	**1991**	**1.56 (1.45–1.67)**	**1.38 (1.32–1.44)**		**1.23 (1.13–1.33)**
Warner et al., 2010^42^	Non-transfused	63	11.7 (7.4)		GOSE 5.7 (2.2)	6 (9.5%)
	**Transfused**	**76**	**23.1 (17.8)**		**3.9 (2.2)**	**13 (17.1%)**
George et al., 2008^46^	Non-transfused	39	13 (9.9)	27 (69%)		11 (29%)
	**Transfused**	43	17.1 (11.7)	36 (85%)		15 (35%)
Leal-Noval et al., 2016^44^	Non-transfused	145	20 (11–34)		Unfavorable GOS 27 (22.0%)	10 (8.1%)
	**Transfused**	**164**	**39 (20–74)**		**64 (46.0%)**	**44 (31.7%)**
Al-Dorzi et al., 2015^45^	Non-transfused	80	54 (73.4)	31 (38.8%)		7 (8.8%)
	**Transfused**	**21**	82 (100.1)	**4 (19.0%)**		8 (38.1%)
Boutin et al., 2018^43^	Non-transfused	149	(ref)	(ref)		(ref)
	**Transfused**	66	**2.13 (1.49–3.05)**	**3.40 (1.35–8.36)**		**2.15 (1.37–3.38)**
Salim et al., 2008^47^	Anemic: non-transfused	126		21 (17%)		17 (13%)
	**Anemic: transfused**	**401**		**114 (28%)**		**119 (30%)**
	Not anemic: non-transfused	495		47 (9%)		56 (11%)
	**Not Anemic: transfused**	**128**		**38 (30%)**		**38 (30%)**

TBI, traumatic brain injury; GOS, Glasgow Outcome Score; GOSE, Glasgow Outcome Score-Extended; LOS, length of stay; SD, standard deviation; IQR, interquartile range; RR, relative risk.

### Comparisons of red blood cell transfusion thresholds

Table 1 summarizes the results of six studies that compared clinical outcomes in TBI patients according to transfusion threshold, along with their levels of evidence. Two studies in this group were RCTs, and they report conflicting results. One RCT, which compared clinical outcomes in patients who were transfused for an hgb of <7 g/dL (restrictive threshold; goal of 7–9) at presentation versus 10 g/dL (liberal threshold; goal of 10–12) at presentation, found a significantly higher rate of thromboembolic events in the liberal threshold group but no significant difference in outcomes.^5^ In contrast, the RCT by Gobatto and colleagues found a higher rate of favorable outcomes and lower rates of mortality and post-traumatic vasospasm, with a transfusion threshold of 9 g/dL compared to 7 g/dL (level 2a).^8^

Another single-center retrospective analysis, which compared outcomes at integral transfusion thresholds between 7 and 10 g/dL, found that each 1-g/dL increase in initial presenting hgb level after trauma improved the probability of a good outcome by 33%.^12^ However, transfusion at presentation for a liberal hgb threshold of <9 or <10 g/dL was associated with worse outcomes, and the authors recommended considering a threshold of ≤8 g/dL based on optimal outcomes (level 2b). Another multi-center study of >1100 patients showed that patients who were transfused at an hgb threshold <10 g/dL had worse 28-day survival and acute respiratory distress syndrome (ARDS)-free survival rates compared to patients who were transfused with an hgb threshold of 7–10 or <7 g/dL.^11^

*Post hoc* analyses of TBI patients from broader RCTs have also been performed. The *post hoc* analysis of TBI patients from the TRICC trial found no significant differences in 60-day all-cause mortality or length of ICU stay between transfusion thresholds of hgb <7 g/dL and an hgb level of <10 g/dL.^5^ However, another analysis of a similar RCT found that the liberal transfusion group had better outcomes in the early post-intervention period, but not long term.^20^ A single-center retrospective comparison of hgb <7- and <10-g/dL transfusion thresholds among 1565 TBI patients by Ngwenya and colleagues found that an hgb goal of >10 was associated with a longer LOS (13.9 vs. 10.9 days),^13^ except among patients with a presenting GCS <8, where there were no differences in LOS, duration on ventilator, or discharge disposition between transfusion thresholds.

### Predictors of need for red blood cell transfusion

Table 2 summarizes the results and levels of evidence of seven studies that compared demographic data between patients who did and did not receive RBC transfusion.^4,14,16–19^ All but one study demonstrated worse GCS on admission for transfused patients, but head AIS at presentation was not significantly associated with transfusion status. Among studies that reported ISS at presentation, all noted greater injury severity in the transfused population, although this difference was not always statistically significant.

### Transfusion-related complications

Table 3 summarizes the results of studies that analyzed transfusion-related complications, with level of evidence found in Appendix A. All included studies determined that patients who undergo RBC transfusion have an increased rate of adverse events.^4,14–19^ This includes an increased relative risk of mortality and LOS,^14,15^ along with increased overall mortality rate,^4,16–19^ worse GOS and GOSE scores at follow-up,^4,16^ and increased risk of sepsis.^3^

### Meta-analysis

**FIG. 2.**
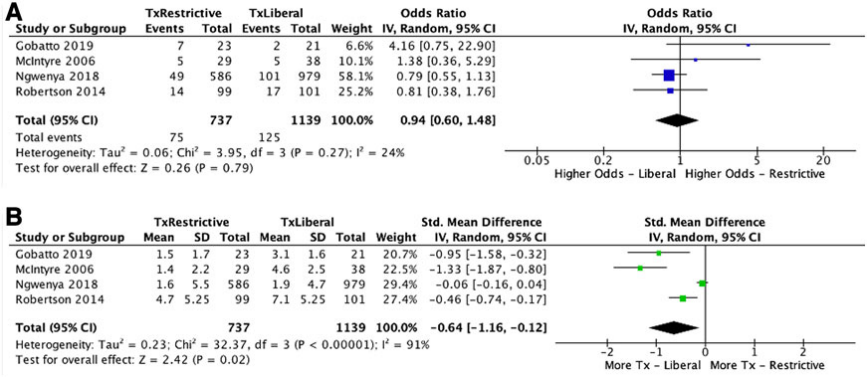
(**A**) Comparison of mortality rate with restrictive and liberal transfusion thresholds. (**B**) Comparison of units of pRBCs transfused with restrictive and liberal transfusion thresholds. 95% CI, 95% confidence interval; IV, inverse variance; pRBCs, packed red blood cells; SD, standard deviation; Tx, treatment.

**FIG. 3.**
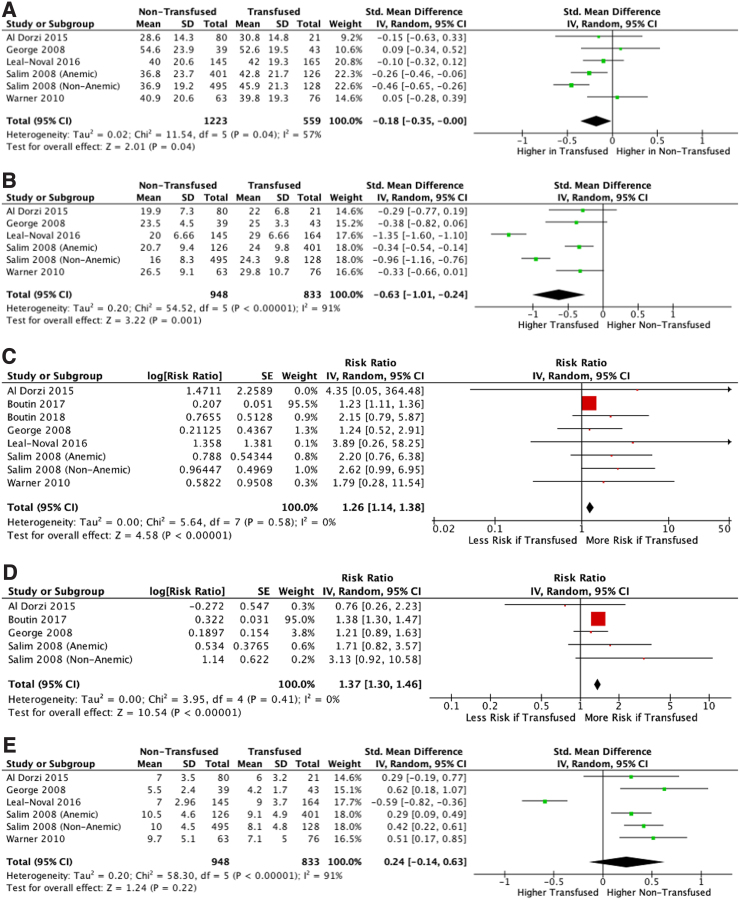
(**A**) Comparison of age in transfused and non-transfused patients. (**B**) Comparison of ISS at presentation in transfused and non-transfused patients. (**C**) Comparison of mortality rate in transfused and non-transfused patients. (**D**) Comparison of complications at presentation in transfused and non-transfused patients. (**E**) Comparison of GCS at presentation in transfused and non-transfused patients. 95% CI, 95% confidence interval; GCS, Glasgow Coma Scale; ISS, Injury Severity Score; IV, inverse variance; SD, standard deviation; SE, standard error; Tx, treatment.

Figures 2 and 3 highlight a meta-analysis of studies included in the systematic review. When comparing patients who did and did not receive transfusions, the primary outcome measures were overall complications and mortality rate. When comparing patients who were transfused at liberal or restrictive thresholds, the primary outcome measures were number of pRBC transfusions and mortality rate. TBI patients who receive transfusions tend to be older (*p* = 0.04; Fig. 3A), have worse ISS scores at presentation (*p* = 0.001; Fig. 3B), and have higher mortality rates (*p* < 0.001; Fig. 3C) and complication rates (*p* < 0.001; Fig. 3D). GCS at presentation was not significantly different between transfused and non-transfused patients (*p* = 0.22). Mortality rate was not significantly different between restrictive and liberal transfusion thresholds (*p* = 0.79; Fig. 2A), and liberal transfusion thresholds led to significantly more units of pRBCs transfused (*p* = 0.02; Fig. 2B).

## Discussion

Transfusion guidelines for TBI patients presents a unique challenge because of the unique oxygenation requirements of cerebral tissue. The TRICC trial, which studied a general trauma patient population and continues to guide current practice, found that a restrictive transfusion threshold of hgb <7 g/dL led to non-inferior outcomes and lower rates of complications compared to a liberal threshold of hgb <10 g/dL.^6^ However, a *post hoc* analysis of TBI patients enrolled in the TRICC showed no difference in outcomes or complications between these thresholds.^5^ To date, our review is the most comprehensive study to analyze outcomes related to transfusion thresholds in TBI patients, as well as the risk factors for transfusion utilization. Based on the currently available data, our meta-analysis found that, despite liberal transfusion groups receiving more units of RBCs, there was no difference in mortality between liberal and restrictive transfusion thresholds. A common confounding variable to these studies is that sicker patients are more likely to receive transfusion, but, interestingly, we found no difference in GCS between transfusion groups. Overall, most previous studies support conservative transfusion protocols, as described in the TRICC trial, but in order for the field to progress toward more definitive guidelines specific to TBI patients, we highlight important nuances in methodology and their implications toward interpretability and extrapolation.

Two previous meta-analyses have been attempted with the primary objective of determining whether the TRICC trial findings remain consistent in the TBI population.^21,22^ Boutin and colleagues, in 2016, primarily analyzed risk factors for transfusion and only reported on outcomes in a qualitative manner because of data sparsity and heterogeneity.^21^ They did not limit their inclusion criteria to only RBC transfusion and included multiple studies analyzing other forms of transfusion. Florez-Perdomo and colleagues, in 2021, conducted a similar study, but their analyses were limited to a small number of studies that fit their narrow inclusion criteria. Curiously, the values utilized in their analyses could not be found in the cited studies, most notably the data on mortality as compared to Table 2 in McIntyre and colleagues and Table 2 in Gobatto and colleagues.^22^ Compared to their meta-analysis of mortality outcomes between liberal and restrictive transfusion groups, our analysis of the same studies found no significant difference between groups (Fig. 2A).

Robertson and colleagues' RCT compared transfusion thresholds of hgb <7 and <10 g/dL, and they found that 42.5% (37 of 87) of patients in the restrictive group had favorable outcomes, compared to 33.0% (31 of 94) of patients in the liberal group, although this difference was not significant.^7^ They also found a significantly higher rate of thromboembolic events in the 7-g/dL group (21.8% vs. 8.1%; 95% confidence interval [CI] difference, 0.12–0.79), and the study had a level of evidence of III B-R. It should be noted, however, that another primary aim of this study was analyzing the effect of erythropoietin in this population, which confounds the conclusions one can draw from this study in regard to transfusion thresholds.

The RCT by Gobatto and colleagues described contrasting results compared to Robertson and colleagues. They found that a transfusion threshold of 9 g/dL was associated with a higher rate of favorable GOS scores compared to a threshold of 7 g/dL.^8^ Of the 21 patients in the 9-g/dL group, there were 2 (9.5%) deaths and 13 (61.9%) patients with favorable GOS scores at 6 months post-op, compared to 7 (30.4%) deaths and 10 (43.5%) patients with favorable GOS scores in the <7-g/dL group. Additionally, they noted a substantial reduction in post-traumatic vasospasm rate for the liberal group (3% vs. 65%) and no difference in mean LOS between groups. Comparatively, a strength of this study is that their only primary outcome was comparing transfusion thresholds, but the main limitation was decreased sample size and subsequent underpowering of their data. The level of evidence for Gobatto and colleagues was IIa B-R.

With the limited data comparing outcomes solely in TBI patients, *post hoc* analyses of larger RCTs provide large portions of data, but they also reinforce the literature's inconsistency with respect to optimal transfusion thresholds in this population. Yamal and colleagues performed a *post hoc* analysis of the study by Robertson and colleagues and found a mortality benefit with the <10-g/dL threshold during the early intervention period.^23^ Litofsky and colleagues analyzed patients with head AIS >3 and compared multiple transfusion thresholds (<7, <8, <9, and <10 g/dL), and they found that whereas patients presenting with lower hgb had worse initial ISS and GCS, transfusion was associated with worse outcomes in the <9- and <10-g/dL groups. The authors suggested that a transfusion threshold of hgb <8 g/dL is optimal for TBI patients, given that it best balances the clinical benefits of increasing hgb with the potential risks of receiving transfusion (IIa B-R). However, given the small number of patients in both of these analyses and the conflicting nature of their findings with other studies, the recommendation to adopt more liberal transfusion thresholds should be taken with careful consideration.

Elterman and colleagues and McIntyre and colleagues performed *post hoc* analyses of the Resuscitation Outcomes Consortium and TRICC trial, respectively, and each found no significant improvement in outcomes with liberal transfusion thresholds (<10 g/dL).^5,11^ McIntyre and colleagues described a non-significant difference in 60-day all-cause mortality between patients who were transfused at hgb <7 g/dL (17%) versus an hgb <10 g/dL (13%), along with non-significant differences in ICU LOS (8 days in the conservative group vs. 10 days in the liberal group; 95% CI, 5–11; *p* = 0.26). Elterman and colleagues found that worse outcomes occurred when transfusing patients with an initial hgb >10 g/dL, including worse 28-day survival and ARDS-free survival post-transfusion.^11^ However, they found no significant difference in outcomes for patients who transfused at hgb <7 g/dL versus those transfused at an hgb of 7–10 g/dL. Elterman and colleagues' and McIntyre and colleagues' studies each carry level III B-R evidence.

Data from other retrospective analyses provide further evidence for caution against liberal transfusion thresholds. Ngwenya and colleagues compared transfusion thresholds of 10 to 7 g/dL among 1565 patients at a single center and found that transfusing at hgb <10 g/dL was associated with a longer LOS on average (13.9 vs. 10.9 days).^13^ However, when limiting the analysis to patients with GCS <8, there was no difference in LOS, duration on ventilator, or discharge disposition. The authors also estimate that the conservative transfusion protocol saved the hospital ∼$115,000 annually. The level of evidence for the study by Ngwenya and colleagues was III C-LD.

Variance in methodology likely accounts for some of the inconsistency in conclusions between studies and may contribute to the lack of a significant difference between liberal and restrictive transfusion thresholds in our meta-analysis. First, Yamal and colleagues nicely highlighted the inconsistencies in study conclusions based on the outcome metrics used.^24^ There is also variation in the thresholds used and how they are implemented. Most studies compare thresholds of 7 and 10 g/dL, but Gobatto and colleagues and Litofsky and colleagues compared intermediate thresholds of 8 and 9 g/dL. In Gobatto and colleagues, hgb was checked at least daily, and RBC transfusion was indicated upon hgb falling below threshold, leading to 100% of patients receiving at least one transfusion at a threshold of 9 g/dL compared to only 57% of those at 7 g/dL. Conversely in Robertson and colleagues, only 72.3% of patients at the threshold of 10 g/dL received a transfusion compared to 52.5% of those at the 7-g/dL threshold.

Differing inclusion criteria also complicates study comparisons. Robertson and colleagues included all patients who had a closed TBI and were unable to follow commands after resuscitation,^7^ whereas Gobatto and colleagues adhered to a more quantitative cutoff of GCS <12 and hgb of <9 g/dL, similar to the original TRICC trial criteria.^5,8^ Given that patients who are able to follow commands are less likely to be suffering from severe cerebral hypoxia, they are less likely to benefit from RBC transfusion and it is important to limit their inclusion in these studies. This is an important nuance, given that multiple studies have suggested that cerebral hypoxia commences at hgb levels at or below 9 g/dL.^25–27^ Oddo and colleagues found that a significant decrease in cerebral oxygenation occurred when hgb dropped to <9 g/dL.^27^ Sekhon and colleagues also found that hgb <9 g/dL was associated with worse clinical outcomes.^28,29^ Yamal and colleagues described cerebral oxygen monitor findings from the Robertson and colleagues trial and found that 25% of patients at the 7-g/dL threshold experienced hypoxic cerebral tissue events compared to only 10.2% in the 10-g/dL group.^23^

Interestingly, this only occurred when probes were placed in healthy cerebral tissue, and there was no difference when they were placed in tissue surrounding the area of contusion. This observation may reflect the pathophysiology underlying ischemic penumbra, whereby the surrounding reversibly injured cerebral tissue remains responsive to RBC transfusion whereas the ischemic core does not. Despite these data that have been replicated multiple times in the literature, it is curious then that only Gobatto and colleagues have sought to perform an RCT comparing the threshold of 9 g/dL.

The goal of transfusion in TBI patients is to prevent secondary ischemic injury of cerebral tissue. RBC transfusion has been repeatedly shown to be an effective treatment for increasing cerebral oxygenation.^20,25,26,30^ However, it is unclear how much of the repleted oxygenation permeates the reversibly injured penumbra versus the rest of the uninjured cerebral tissue, and, moreover, RBC transfusions come with well-documented risks. The ideal transfusion threshold must maximize the clinical benefits while limiting exposure to the potential negative side effects. Based on past data, it is possible that the transfusion threshold of hgb <10 g/dL may include patients who are at minimal risk of cerebral hypoxia and thus may be unnecessarily exposed to the risks of transfusion. Additionally, establishing an optimum transfusion threshold in TBI patients is important, because there are few other effective strategies for promoting recovery of oxygen-carrying capacity. Erythropoietin administration can increase circulating hgb by stimulating *de novo* RBC proliferation, but its utility in the TBI population is unclear. Despite initial negative findings by Robertson and colleagues, a *post hoc* analysis of their data by Benoit and colleagues noted an improvement in longitudinal outcomes for severe TBI patients with erythropoietin administration.^31^

### Demographic and comorbid predictors of red blood cell transfusion

Quantifying predictors of RBC transfusion in the TBI population found that they are consistent with those reported for the greater critical care population.^32^ Multiple studies suggest that transfused populations have worse injury severity as demonstrated by the GCS, head AIS, and head ISS indices.^3,14,15,18,19^ Warner and colleagues also noted that transfusion status and GCS on admission were independent predictors for long-term functional outcome after multi-variate regression.^4^ AIS and ISS may predict clinical outcome at 12 months,^33^ and it is important to consider these associations in the context of transfusion threshold analyses, given that outcomes between groups may simply be the result of transfused patients being sicker and more likely to receive transfusion. It is curious that, although ISS was worse in transfused groups as expected, our meta-analysis found no difference in GCS between transfusion groups. One potential explanation is that this finding highlights the heterogeneity in results based on the metrics utilized, similar to the outcomes data described by Yamal and colleagues.

### Transfusion-related complications

Avoidance of transfusion-related complications is an essential consideration when developing a transfusion threshold. The mortality rate in transfused TBI patients ranged from 17.1% to 38.1%, with Boutin and colleagues and George and colleagues reporting increased relative risks for mortality of 1.23 (95% CI, 1.13–1.33) and 2.15 (95% CI, 1.37–3.33), respectively.^14,18^ Conversely, the mortality rates for non-transfused patients were under 10% in all studies, aside from George and colleagues and Salim and colleagues.^18,19^ Complications were also often worse in the transfused groups. Boutin and colleagues reported a relative risk of 3.40 (95% CI, 1.35–8.36) for neurological complications in the transfused population,^34^ and Salim and colleagues noted worse overall complication rates with transfusion among anemic patients.^19^ However, there were no statistically significant differences in ARDS, acute renal failure, multi-system organ failure, or pulmonary embolism based on transfusion status,^19^ and Al-Dorzi and colleagues did not report any difference in complications between transfusion groups.^17^ Our meta-analysis determined that transfused patients have higher rates of mortality, which likely suggests that patients who require a transfusion have an inherently worse prognosis at baseline, but further clarification is warranted given the heterogeneity of included studies.

### Limitations

The current systematic review is limited by the quality and consistency of the included studies. First, inclusion criteria are inconsistent regarding the definition of TBI, whether anemic versus non-anemic patients are eligible for inclusion, follow-up time, and the specific definitions “restrictive” and “liberal” transfusion thresholds. Variation in these parameters impart heterogeneity to a meta-analysis of pooled data, and these parameters should be standardized in future studies. Additionally, only two included studies were RCTs that sought to directly compare outcomes in TBI patients between transfusion thresholds. The remaining studies were *post hoc* analyses of RCTs or unmatched retrospective analyses, which are more subject to selection and confounding biases. To definitively address the question of optimum transfusion threshold for TBI patients, we advocate for a multi-institutional, prospective RCT that is appropriately powered to differentiate between multiple transfusion thresholds.

## Conclusion

Current literature appears to support restrictive transfusion protocols (hgb <7 g/dL) compared to liberal thresholds (hgb <10 g/dL) after TBI, but the quality and consistency of evidence is mixed. The best data can be derived from two RCTs and multiple *post hoc* analyses of RCTs not limited to the TBI population, but there is significant variance in methodology between these studies. Standardized criteria regarding the definition of TBI severity, inclusion of anemic patients, hgb monitoring, indications for transfusion, and consideration of the unique oxygenation requirements of cerebral tissue are important foundations on which to build future studies to more definitively answer this important question.

## Abbreviations Used

ACC: American College of Cardiology
AHA: American Heart Association
AIS: Abbreviated Injury Score
ARDS: acute respiratory distress syndrome
CI: confidence interval
GCS: Glasgow Coma Score
GOS: Glasgow Outcome Score
GOSE: Glasgow Outcome Score-Extended
hgb: hemoglobin
ICU: intensive care unit
ISS: Injury Severity Score
LOS: length of stay
OR: odds ratio
pRBC: packed red blood cell
PRISMA: Preferred Reporting Items for Systematic Reviews and Meta-Analyses
RBC: red blood cell
RCT: randomized controlled trial
SMD: standardized mean difference
TBI: traumatic brain injury

## Appendix

**Appendix Table AT1.** Summarizing the Results of All Studies Included in the Systematic Review

Author, year, title, and location	Study type	Study aim	Study conclusions	Limitations	AHA level of evidence classification
Al-Dorzi et al., 2015. Anemia and Blood Transfusion in Patients with Isolated Traumatic Brain Injury. Saudi Arabia, Lebanon.^45^	Retrospective cohort	To determine anemia incidence in patients with isolated TBI during ICU stay, describe blood transfusion practices in them, and assess the impact of anemia and blood transfusion on their outcomes	Packed RBC transfusion independently predicted mortality.	Lack of data on daily fluid balance and the age of RBCs, which has been said to affect the outcome of patients	Class C-LD, level III, harm
Boutin et al., 2017. Transfusion of red blood cells in patients with traumatic brain injuries admitted to Canadian trauma health centres. Canada.^41^	Retrospective case control	To evaluation the frequency of RBCT, determinants of transfusion, and associated clinical outcomes	RBCT is associated with unfavorable outcomes.	Some patients missing GCS, RBCT events, and hgb levels during hospitalization	Class C-LD, level III, risk
Boutin et al., 2018. Hemoglobin thresholds and red blood cell transfusion in adult patients with moderate or severe traumatic brain injuries. Canada.^43^	Retrospective cohort	To describe pre-transfusion hgb thresholds and RBC use in critically ill patients with TBI and estimate the association between pre-transfusion hgb levels and transfusion practices and clinical outcomes	During ICU stay, transfused patients tended to have lower hgb levels and worse outcomes than patients who did not receive RBCs.	Missing data on timing of surgical interventions, timing of neurological and/or trauma complications	Class C-LD, level III, benefit
Elterman et al., 2013. Transfusion of red blood cells in patients with a prehospital Glasgow Coma Scale score of 8 or less and no evidence of shock is associated with worse outcomes. USA, Canada.^35^	*Post hoc* review of secondary outcomes of RCT	From a multi-center randomized controlled trial evaluating early use of hypertonic fluids to restore cerebral perfusion, this study analyzed the association of RBCT and outcomes.	In patients with a suspected TBI and no evidence of shock, transfusion of RBCs was associated with worse outcomes when the initial hgb was >10.	GCS is a limited predictor of TBI; 29% of patients lacked an anatomical finding of TBI and inconsistent indication for transfusion.	Class B-R, level III, risk
George et al., 2008. Aggressive Red Blood Cell Transfusion: No Association with Improved Outcomes for Victims of Isolated Traumatic Brain Injury. USA.^46^	Retrospective case control	To test the hypothesis that patients with severe isolated TBI would not benefit from traditional aggressive RBCT	Our results suggest that a restrictive transfusion practice is safe for severely TBI-injured patients.	Low sample size, missing data on post-procedural blood loss, and management of TBI has undergone changes since completion of this study.	Class C-LD, level III, risk
Gobatto et al., 2019. Transfusion requirements after head trauma: a randomized feasibility controlled trial. Brazil.^39^	Randomized controlled trial	Pilot randomized controlled trial directly comparing liberal and restrictive hgb thresholds for transfusion	Hospital mortality was lower, and neurological status at 6 months favored the liberal group.	Small sample size and decreased recruitment attributable to requirement of consent	Class B-R, level IIa, benefit
Leal-Noval et al., 2016. Effects of Red Blood Cell Transfusion on Long-Term Disability of Patients with Traumatic Brain Injury. Spain.^44^	Prospective case control	To investigate the influence of RBCT on the functional status of patients with TBI	RBCT may be associated with worsened long-term neurological outcomes in patients with TBI.	Loss of patients during study and failure to account for important interaction among time, RBCT, and hgb concentration	Class B-NR, level III, harm
Litofsky et al., 2016. The Negative Impact of Anemia in Outcome from Traumatic Brain Injury. USA.^36^	Retrospective cohort	To compare survival and neurological outcomes between different hgb thresholds in TBI patients	Outcome after TBI is worse in patients with lower hgb. The data support the consideration of blood transfusion when hgb is <8 g/dL.	Insufficient detailed medical records and variation in patient follow-up	Class C-LD, level IIb, benefit
McIntyre et al., 2006. Effect of a Liberal Versus Restrictive Transfusion Strategy on Mortality in Patients With Moderate to Severe Head Injury. Canada.^40^	*Post hoc* subgroup analysis of RCT	*Post hoc* analysis of TBI patients from the TRICC trial comparing hgb thresholds for transfusion	No significant improvements in mortality with a liberal transfusion strategy in critically ill trauma victims with moderate-to-severe TBI	Wide confidence intervals and small sample size	Class B-R, level III, risk
Ngwenya et al., 2018. Safety and cost efficiency of a restrictive transfusion protocol in patients with traumatic brain injury. USA.^37^	Retrospective case control	To evaluate the safety and cost-efficiency of hospital-wide adherence to a restrictive transfusion threshold	The benefits of restrictive transfusion outweigh the risks; the transfusion threshold of hgb <7 g/dL should be standard policy for patients with TBI.	Patient populations that may need a more aggressive transfusion protocol because of medical comorbidities not addressed	Class C-LD, level III, risk
Okoye et al., 2013. The impact of anemia in moderate to severe traumatic brain injury. USA.^48^	Retrospective cohort	To examine the outcome of varying degrees of anemia in patients who have sustained a TBI	Anemia in TBI patients as low as 8 g/dL was not associated with increased mortality or complications, whereas transfusion was associated with an increase in septic complications.	Does not report neuro outcome and no rationale for transfusing non-anemic patients	Class C-LD, level III, risk
Robertson et al., 2014. Effect of Erythropoietin and Transfusion Threshold on Neurological Recovery After Traumatic Brain Injury—Randomized Clinical Trial. USA.^38^	Randomized controlled trial	Randomized controlled trial evaluating effects of erythropoietin on outcomes in TBI patients, while simultaneously comparing transfusion thresholds	Maintaining an hgb concentration of >10 g/dL did not result in improved neurological outcome at 6 months, but was associated with a higher incidence of adverse events.	Trial was only conducted at two sites, which could limit generalizability of results	Class B-R, level III, risk
Salim et al., 2008. Role of Anemia in Traumatic Brain Injury. USA.^47^	Retrospective cohort	To test the hypothesis that anemia in TBI patients would be associated with unfavorable outcomes	Blood transfusion is associated with significantly worse outcomes in TBI patients.	None listed by the authors	Class C-LD, level III, harm
Warner et al., 2010. Transfusions and long-term functional outcomes in traumatic brain injury. USA.^42^	Retrospective case control	To examine the relationship between transfusion and long-term functional outcomes in moderately anemic patients with TBI	Transfusions may contribute to poor long-term functional outcomes in anemic patients with TBI.	Transfused group had worse GCS than non-transfused and unsure of indications for transfusion	Class C-LD, level III, harm
Yamal et al., 2015. Effect of Hemoglobin Transfusion Threshold on Cerebral Hemodynamics and Oxygenation. USA.^49^	Retrospective case control	To examine the secondary outcome measures of intracranial pressure, cerebral perfusion pressure, and brain tissue oxygenation	These data do not support the use of a higher transfusion threshold even in the early resuscitation period.	Missing values of hemodynamic outcomes and measurement bias	Class C-LD, level III, risk

RCT, randomized controlled trial; TBI, traumatic brain injury; ICU, intensive care unit; RBC, red blood cell; RBCT, red blood cell transfusion; hgb, hemoglobin; GCS, Glasgow Coma Scale.
